# Optimizing methanol synthesis from CO_2_ using graphene-based heterogeneous photocatalyst under RSM and ANN-driven parametric optimization for achieving better suitability

**DOI:** 10.1039/d4ra00578c

**Published:** 2024-04-17

**Authors:** Ramesh Kumar, Jayato Nayak, Somnath Chowdhury, Sashikant Nayak, Shirsendu Banerjee, Bikram Basak, Masoom Raza Siddiqui, Moonis Ali Khan, Rishya Prava Chatterjee, Prashant Kumar Singh, WooJin Chung, Byong-Hun Jeon, Sankha Chakrabortty, Suraj K. Tripathy

**Affiliations:** a Department of Earth Resources & Environmental Engineering, Hanyang University 222-Wangsimni-ro, Seongdong-gu Seoul 04763 Republic of Korea bhjeon@hanyang.ac.kr; b Centre for Life Science, Mahindra University Hyderabad Telangana 500043 India; c Department of Chemical Engineering, NIT Durgapur M.G. Avenue 713209 West Bengal India; d School of Chemical Technology, Kalinga Institute of Industrial Technology Bhubaneswar Odisha India-751024 sankha.chakrabortty@kiitbiotech.ac.in; e Centre for Creative Convergence Education, Hanyang University 222 Wangsimni-ro, Seongdong-gu Seoul 04763 Republic of Korea; f Petroleum and Mineral Research Institute, Hanyang University 222 Wangsimni-ro, Seongdong-gu Seoul 04763 Republic of Korea; g Chemistry Department, College of Science, King Saud University Riyadh 11451 Saudi Arabia; h RCI Office, National Institute of Technology Durgapur M.G. Road Durgapur 713209 West Bengal India; i Department of Biochemistry, University of Lucknow Lucknow-226007 Uttar Pradesh India; j Department of Environmental Energy Engineering, Kyonggi University Suwon 16227 Republic of Korea cine23@kyonggi.ac.kr

## Abstract

Assessment of the performance of linear and nonlinear regression-based methods for estimating *in situ* catalytic CO_2_ transformations employing TiO_2_/Cu coupled with hydrogen exfoliation graphene (HEG) has been investigated. The yield of methanol was thoroughly optimized and predicted using response surface methodology (RSM) and artificial neural network (ANN) model after rigorous experimentation and comparison. Amongst the different types of HEG loading from 10 to 40 wt%, the 30 wt% in the HEG-TiO_2_/Cu assisted photosynthetic catalyst was found to be successful in providing the highest conversion efficiency of methanol from CO_2_. The most influencing parameters, HEG dosing and inflow rate of CO_2,_ were found to affect the conversion rate in the acidic reaction regime (at pH of 3). According to RSM and ANN, the optimum methanol yields were 36.3 mg g^−1^ of catalyst and 37.3 mg g^−1^ of catalyst, respectively. Through the comparison of performances using the least squared error analysis, the nonlinear regression-based ANN showed a better determination coefficient (overall *R*^2^ > 0.985) than the linear regression-based RSM model (overall *R*^2^ ∼ 0.97). Even though both models performed well, ANN, consisting of 9 neurons in the input and 1 hidden layer, could predict optimum results closer to RSM in terms of agreement with the experimental outcome.

## Abstract 2

Assessment of the performance of linear and nonlinear regression-based methods for estimating *in situ* catalytic CO_2_ transformations employing TiO_2_/Cu coupled with hydrogen exfoliation graphene (HEG) has been investigated.

## Graphical abstract

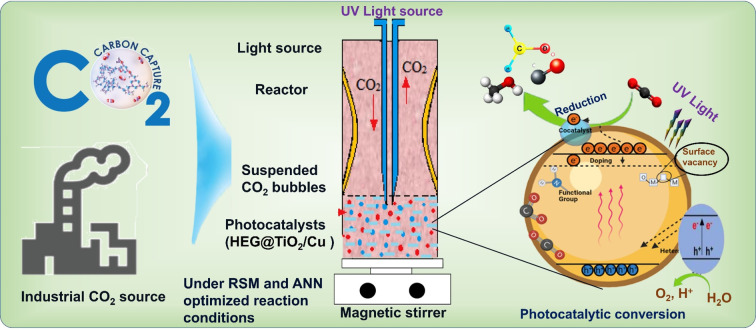

## 1. Introduction

Amidst the worldwide growing energy crisis, fixed and dwindling stock of fossil fuels, and extreme pollution, finding acceptable means to produce alternative fuels is a significant breakthrough.^1,2^ According to experts, the exponential growth in atmospheric CO_2_ concentration caused by human activity is the most severe threat biotic societies face nowadays. Human population expansion, a modern luxurious lifestyle, and significant industrial development have all increased CO_2_ emissions, making this an increasingly serious issue.^3^ There is no better method for addressing the energy and environmental crises than by utilizing cutting-edge photocatalytic technology to convert ambient CO_2_ into useable fuel hydrocarbons (such as methanol or ethanol) under solar excitation.^4,5^ Next-generation biomimetic technologies look promising because they reduce potential pollutants while also converting to low-cost hydrocarbon-based fuels, *viz.*, methanol, by using solar energy and atmospheric CO_2_ as raw materials.^6^ The development of appropriate photocatalysts for the effective redox photosynthesis of CO_2_ to hydrocarbons under UV/visible light, on the other hand, remains a considerable challenge.^7,8^ Semiconductors such as titanium dioxide (TiO_2_) have gained more attention among researchers due to their possible photocatalysis applications. When exposed to ultraviolet light, which accounts for only about 45 percent of total solar radiation, TiO_2_ with a large band gap (about 3–3.2 eV) becomes active.^6^ Metal and non-metal doping of responsive photocatalytic systems^9,10^ and carbon changes that promote electron–hole pair recombination have been extensively investigated to utilize solar visible light irradiation efficiently.

In recent years, mathematical tools for modeling have steadily grown in optimizing bioproduct synthesis and resource recovery.^11,12^ Response surface methodology (RSM) and artificial neural networks (ANN) are two examples of complex mathematical methodologies to elucidate predictive analysis.^13^ If the physical meaning of the system or process under investigation is not explicitly stated, neither methodology nor the scientific method is required.^11^ Thus, both RSM and ANN are modeling approaches concerned with producing flexible and faster non-parametric simulative models instead of parametric models.^14,15^ ANN has been used widely by researchers in a variety of applications related to energy generation, including biomass, wind, hydropower, geothermal, and solar.^16^ An ANN approach was successfully implemented in magnetic field-influenced water electrolysis to predict the hydrogen evaluation with a mean squared error (MSE) of 0.0112 and a correlation coefficient of 0.97.^17^ An optimized numerical matrix can be formulated without any assumptions by RSM predicting the best possible outcome. By considering interactions among different numbers and sets of influential variables, RSM works with fewer experimental trials for validation, unlike ANN.^18^ Due to the advanced genetic algorithm coupled with neurons and hidden layers in the ANN, it can compute and train even with many experimentally obtained nonlinear data.^17^ Thus, the predictive analytical outcome from ANN was generally more refined and accurate than RSM. Both models can be applied in several scientific domains to use experimental data to determine the functional relationships between the process's input variables and their output response variables.^18^ Once this is completed, the models may be used to identify which input variables should be used to achieve the best possible result. Much research employed the evolutionary algorithm in conjunction with ANN to find the best feasible operational variables for the process under consideration.^16^ The RSM and ANN-based optimizations on different feedstocks were described mainly in the literature, and conventional, ultrasonic, and photocatalytic methodologies were used to achieve the best results.^19,20^

The research aims to bridge a significant gap in achieving maximum methanol yield under a maximum number of optimized operational conditions by conducting a comparative analysis of the performance of graphene-coupled Cu/TiO_2_ heterogeneous catalysts using RSM and ANN optimization techniques. The primary objective is to showcase the efficient conversion of CO_2_ into methanol through photocatalysis, leveraging parametric optimization strategies. RSM acts as a statistical tool to analyze the impact of various variables on methanol yield while employing a desired function method alongside optimization techniques to establish an optimized operational framework. Moreover, the study delves into the ANN's capability to predict methanol yield through both predictive and generalized approaches. Evaluation metrics such as coefficient of determination (*R*^2^), root mean square error (RMSE), and *d*-Willmott index are utilized to assess the effectiveness of both models in validating datasets. This comprehensive approach aims to shed light on the efficacy of the catalyst and optimization techniques in enhancing methanol production. The study's findings are expected to advance the understanding of CO_2_ conversion technologies significantly and offer valuable insights for designing more efficient photocatalytic systems for methanol synthesis.

## 2. Materials and methods

### 2.1 Chemicals and preparation of photocatalysts

For developing hydrogen exfoliation graphene (HEG) and graphene embedded catalysts, such as HEG@TiO_2_/Cu, all the materials such as graphite flakes, *N*-methyl pyrrolidone, H_2_SO_4_, KMnO_4_, NaNO_3_, CuSO_4_, and photocatalytic grade TiO_2_ were procured reagent grade chemicals from Sigma-Aldrich, (USA) and Sisco Research Laboratory (India). All the solutions were prepared using Milli-Q water from Merck-Millipore (USA).

The details of the photocatalyst preparation and its characterization were found in our previous work.^4^ The method of photocatalyst preparation is described here briefly. The graphene oxide (GO) was prepared using a modified Hammer's method.^21^ The indigenously prepared GO was employed as the primary feedstock for the hydrogenation process for 2–3 min at the temperature range of 180 to 200 °C to produce porous HEG. The HEG and copper-loaded TiO_2_ were synthesized using a typical sol–gel process in an ultrasonication environment, and the resulting coupling was recovered and studied. The following steps were carried out: (i) a beaker containing 30 mL of absolute ethanol was sonicated in an ultrasound bath for 30 min; (ii) a binder agent containing 15 mL of glucose solution (0.01 M) was added to the alcoholic solution; (iii) copper salt was added; and finally, (iv) two different weight percent HEG was added to the solution and sonicated for 2 hours. The prepared photocatalysts were settled in 12 h and dried at 85 °C for 12 h. The dried powder was roasted in a muffle furnace for 2 h at 500 °C. The roasted powder was crushed and sieved (40–60 mesh size) for CO_2_ reduction experiments.

### 2.2 Experimental setup and procedure

**Fig. 1:**
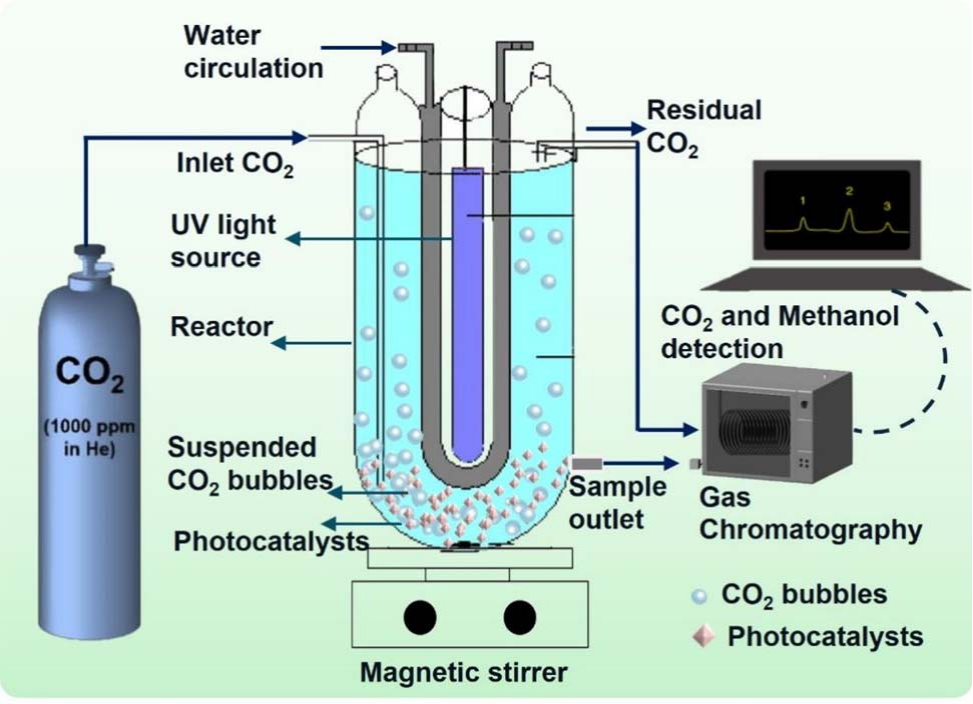
Schematic diagram of the experimental setup of the APQ reactor fitted with CO_2_ cylinder for methanol conversion.

The photocatalytic reduction experiments were conducted in batch mode using a custom-designed annular-type Pyrex quartz reactor (APQ), as depicted in Fig. 1.^4^ Equipped with a 250 W UV light source, the APQ reactor facilitated the photocatalytic process. To introduce CO_2_ into the system, a single tube connection linked the APQ reactor to a CO_2_ cylinder. Moreover, an input–output water connection was integrated into the reactor to regulate its internal temperature. Before UV light irradiation, certified supercritical fluid-grade carbon dioxide was purged into the solvent, typically ultrapure water, for 30 min. Following this, the CO_2_ purging was ceased. The synthesized catalyst, HEG-TiO_2_–CuSO_4_, underwent immersion in 500 mL of ultrapure water and subsequent purification with CO_2_ prior to its introduction into the solution. This meticulous procedure ensured the preparation of the catalyst under controlled conditions, thereby optimizing its performance for subsequent experimentation. By adhering to stringent protocols for catalyst preparation and experimental setup, the study aimed to ensure reproducibility and reliability in the obtained results. Such attention to detail not only enhances the validity of the findings but also contributes to the advancement of knowledge in photocatalytic CO_2_ reduction processes. The investigation delved deeply into the multifaceted effects of various operational parameters on the experimental outcomes. Parameters such as CO_2_ flow rate, temperature, pH, catalyst dose, HEG ratio, stirring time, and operation time were meticulously studied. The CO_2_ flow rate, crucial for the carbon source, was systematically varied from 0.5 to 5 L min^−1^ to assess its impact on methanol yield and reaction kinetics. Temperature variations, ranging from 25 to 55 °C, were explored to understand their influence on reaction rates and catalyst activity. The pH of the reaction medium, adjusted between 2 and 10, played a pivotal role in governing surface charge distribution, thereby affecting catalyst performance and selectivity. Catalyst dose, ranging from 2 to 12 g L^−1^, was optimized to strike a balance between maximizing active sites for catalysis and minimizing mass transfer limitations.

Additionally, stirring time and stirring speed, ranging from 30 to 300 min and 100 to 300 rpm, respectively, were investigated to ascertain their roles in enhancing mass transfer and reaction kinetics. The properties of the developed material underwent rigorous validation through an extensive array of characterizations, encompassing a comprehensive suite of tests and analyses. These characterizations, as outlined meticulously in our previous study,^4^ included examinations of morphological structure, crystallinity, functional group composition, and specific surface area. Such exhaustive characterizations ensured a thorough evaluation of the material's structural, mechanical, and chemical attributes. This comprehensive approach not only elucidated the complex interplay between operational parameters and experimental outcomes but also provided a robust foundation for understanding the material's properties and performance, thus contributing significantly to advancing the field's knowledge base.

### 2.3 Analytical determination

A gas Chromatograph (GC, Agilent Technologies Pvt. Ltd) was used to measure the methanol in the samples. The detection procedure used a flame ionization detector (FID), a DB-Wax column (30 m × 0.25 mm, and 0.25 μm), and helium as the carrier gas. The samples were prefilter with 0.22 μm disc filter (PTFE) to remove the suspended particles before injecting them into the GC for analysis. The following equation was used to compute the yield of methanol produced:1
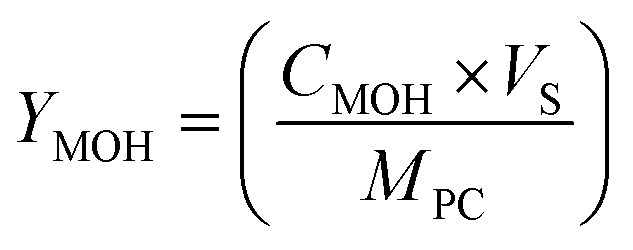
where *Y*_MOH_ is the methanol yield in μM g^−1^ of catalyst, *C*_MOH_ is the methanol concentration in μM L^−1^, *V*_S_ is the volume of reactant solution in L, and *M*_PC_ is the mass of photocatalyst in g.

### 2.4 Design of experiment using response surface methodology

RSM is one of the most popular statistical tools for optimizing governing variables while developing various multi-variant mathematical equations. It is a statistical method that helps several design parameter values to obtain maximum output results while enhancing the overall process to predict optimum values of design experiments based on a minimum set of experiments for the maximization of outcome.^22,23^ The objective of RSM is to use a series of design experiments to achieve an optimum response to the process affected by various input variables. Among the different RSM techniques, central composite design (CCD) is the most popular technique used in design experiments.^24,25^

All experiments were executed according to the statistical design as prepared by Design Expert software (Version 11.0, Stat-Ease, Minneapolis, USA). Five input parameters, such as GO load, stirring speed, CO_2_ flow, time, and pH, were varied, and methanol yield was considered as the output or response variable. The input variables' minimum (−1) values were 10%, 2 g L^−1^, 1 L min^−1^, 30 min, and 1 for GO load, stirring speed, CO_2_ flow, time, and pH, respectively. The input parameters' maximum (+1) values were 50%, 18 g L^−1^, 5 L min^−1^, 150 min, and 5 for GO load, catalyst dose, CO_2_ flow, time, and pH, as shown below in Table 1. The CCD of experiments done in Design Expert software is shown in Table 2. The design experiment was performed in the model based on several input parameters. All the process variables were essential for the experimental design approach as they are linked.

**Table 1:** All coded factor values for methanol yield

Factor	Name	Units	Minimum	Maximum	Coded low	Coded high
*A*	Graphene oxide dose	%	10	50	20	40
*B*	Stirring speed	rpm	100	300	150	250
*C*	CO_2_ flow rate	L min^−1^	1	5	2	4
*D*	Time	min	30	150	60	120
*E*	pH	—	1	5	2	4

**Table 2:** Statistical experimental design for RSM and ANN model

HEG loading (%) [*A*]	Stirring speed (rpm) [*B*]	CO_2_ flow rate (L min^−1^) [*C*]	Time (min) [*D*]	pH [*E*]	Observed methanol yield (yield_RSM/ANN,exp_) (mg g_cata_^−1^)	RSM predicted methanol yield (yield_RSM,model_) (mg g_cata_^−1^)	ANN predicted methanol yield (yield_ANN,model_) (mg g_cata_^−1^)
30	200	3	90	3	38	36.3	37.304
30	200	1	90	3	29	27.8	33.915
30	200	3	90	1	8	7.7	9.171
30	200	5	90	3	36	35.1	32.522
40	150	2	120	4	26	24.3	27.493
40	150	4	60	2	24	23.4	24.044
40	250	2	60	4	25	24.1	24.256
30	200	3	90	3	38	36.2	37.304
30	200	3	30	3	37	35.6	36.738
40	150	4	120	2	26	24.6	22.551
10	200	3	90	3	15	13.8	14.505
20	150	2	120	2	10	9.1	10.503
20	150	2	120	4	8	6.9	9.074
30	200	3	90	3	38	36.3	37.304
20	250	4	120	2	8	7.0	8.631
40	250	4	60	2	16	14.2	15.735
40	250	4	120	4	23	22.4	20.695
30	200	3	90	3	37	35.4	37.304
40	150	2	120	2	18	16.2	17.596
30	200	3	150	3	32	29.4	35.598
40	250	4	60	4	26	24.2	25.623
20	150	2	60	2	12	10.2	12.612
20	150	4	120	2	10	9.1	9.629
50	200	3	90	3	30	28.2	29.728
30	200	3	90	3	38	36.3	37.304
20	150	4	60	2	20	18.4	13.495
20	150	4	120	4	11	9.3	8.588
40	250	2	60	2	14	12.4	14.191
30	100	3	90	3	14	12.3	14
30	200	3	90	3	38	36.3	37.304
20	250	4	60	2	15	12.9	14.843
30	200	3	90	3	38	36.3	37.304
30	200	3	90	5	18	16.4	14.405
40	250	2	120	4	25	23.4	24.366
40	150	4	120	4	27	25.2	26.792
30	300	3	90	3	17	15.5	16.091
40	150	2	60	4	22	21.5	21.53
40	250	4	120	2	19	18.4	19.195
40	150	4	60	4	22	20.7	22.061
20	150	4	60	4	10	8.8	9.514
30	200	3	90	3	38	36.25	37.304
40	250	2	120	2	12	11.1	18.607
20	250	4	120	4	13	11.7	13.159
20	250	2	120	2	9	8.2	9.757
20	250	2	60	2	15	13.9	16.455
20	250	2	120	4	18	16.5	17.222
20	250	2	60	4	21	20.2	22.145
20	150	2	60	4	10	9.3	10.258
20	250	4	60	4	20	19	20.275
40	150	2	60	2	18	16.6	18.443

Among different RSM techniques, the CCD technique is the most suitable for the design of experiments.^23,26^ It mainly contains 2^*n*^ factorial runs with 2*n* axial runs and *n*_*c*_ centre runs. Number of experiments various process variables can be computed as follows,

2 *N* = 2^*n*^ + 2 × *n* + *c* = 2^5^ + (2 × 5) + 8 = 50

where *N* is the number of experiments at runs required, and *n* is the number of independent variables, whereas *c* is the central point. The CCD technique contains three processes: performing design experiments, calculating the model coefficients, and anticipating the behavior and acceptance of the model. Therefore, an empirical model is established to calculate the behavior as a function of various input process parameters and their relations. Finally, the quadratic regression model equation is developed to realize the activities of the process.3
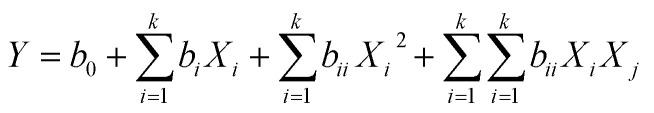
where *i* and *j* are linear and quadratic coefficients, respectively, *b*_0_ is a constant, *b*_*i*_ is a linear coefficient, *b*_*ii*_, and *b*_*ij*_ represent interactive and quadratic coefficients, respectively.^24,27^ The acceptability of the suggested polynomial model equation was evaluated by the values of *R*^2^, *R*_Adj_^2^ and *R*_Pred_^2^. When the values of correlation coefficient were higher, the fitting of experimental data was better than the suggested polynomial equation.^28^

### 2.5 Design of experiment using artificial neural network

ANN is a deep learning technology similar to biological nervous systems. The advanced data modeling method utilizes the structural and functional capabilities of the nervous systems, teaching them the computation of the predicted values from the input data. The model characterizes the nonlinear relationship between the input and the output variables functioning to understand the underlying representation of the same. The multi-layer perceptron (also known as neural networks) is represented mathematically by three layers operating in parallel: input, hidden, and output layers. The data is fed to the input layer (*X*), where it is multiplied with the scalar weights (*w*) and summed with biases (*b*). Weights and biases are the adjustable parameters of the model and control the influence of the processing in the form of signals.^29,30^ The idea of artificial intelligence that formulates the relationship between the human brain and the nervous system was used to develop the ANN model.^31,32^ The following function (*wX* + *b*) is transferred to the activation function in the hidden layers where non-linearity is introduced. Designing a neural network model is pivotal in optimizing and fine-tuning the hyperparameters, which is crucial for generating network outputs and errors. In the post-training process, the model outputs are compared with the target vectors, leading to the computation of error by difference. With the assistance of the backpropagation algorithm, these errors are back-tracked to update the weights and biases for efficient performance.^33,34^ There are several advantages of the ANN model over traditional mathematical models, such as a detailed understanding of the process, which may not be needed, and the fact that it can be developed fast based on process input and outputs in relatively less time than rigorous mathematical models. The backpropagation algorithm is a supervised learning method that minimizes errors between the predicted outputs and experimental data. Levenberg–Marquardt's backpropagation algorithm with gradient descent optimization was employed in the present study. This algorithm has been one of the most common and highly utilized processes for ANN design.

In this study, an experimental database includes 50 data on methanol synthesis. The detailed statistics of the experimental tests database are shown in Table 1. The input parameters for obtaining an appropriate network are GO load, catalyst dose, CO_2_ flow rate, time, and pH. The Levenberg–Marquardt method was utilized to train the algorithm. The input and output are randomly sampled into three sets: training, testing, and validation for constructing the network using the Levenberg–Marquardt algorithm.

A meticulous data management strategy was implemented to ensure robust model training and evaluation. The dataset was partitioned based on percentages, following the guidelines proposed by Hastie *et al.*, with 60% allocated for training, 20% for validation, and the remaining 20% reserved for rigorous testing of the developed neural network model.^35^ This approach adheres to best practices in machine learning, allowing for an adequate amount of data to train the model while also providing distinct subsets for validation and testing. The 60% training set facilitates the network's learning process by exposing it to a substantial portion of the available data, enabling it to capture underlying patterns effectively. The 20% validation set serves as a means to fine-tune model hyperparameters and prevent overfitting, ensuring that the network generalizes well to unseen data. Finally, the remaining 20% of the data is reserved exclusively for testing, serving as an independent benchmark to assess the model's performance objectively. This meticulous partitioning strategy enhances the credibility and reliability of the study's findings, as it minimizes the risk of overfitting and ensures that the model's performance is accurately assessed across diverse datasets. By adhering to such rigorous data management practices, the study maintains scientific integrity and enhances the reproducibility of its results. The training set is used for adjusting connected weights and biases, the validation set is used for checking overfitting problems, and the testing set is used for checking network performance after training. Overtraining must be avoided as it will lead to poor predictive performance and is indicated by increased validation error.^36^ The requirement of hidden neurons plays an indispensable role in ANN training. Therefore, the exact number of hidden neurons must be calculated beforehand, as too few or too many hidden neurons will result in poor performance and overtraining. The input and target parameters were normalized to enhance the network's performance using the max–min method between 0 and 1.

Coefficient of determination4
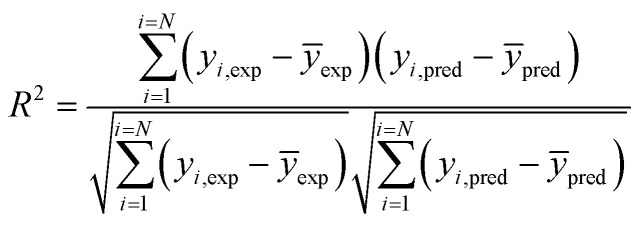


Mean square error5
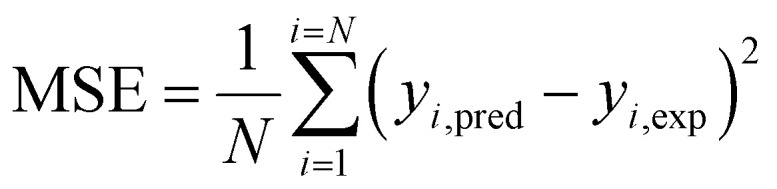
where, *y*_*i*,pred_ is predicted output and *y*_*i*,exp_ is experimental or target output, and *N* is the number of data points.

**Fig. 2:**
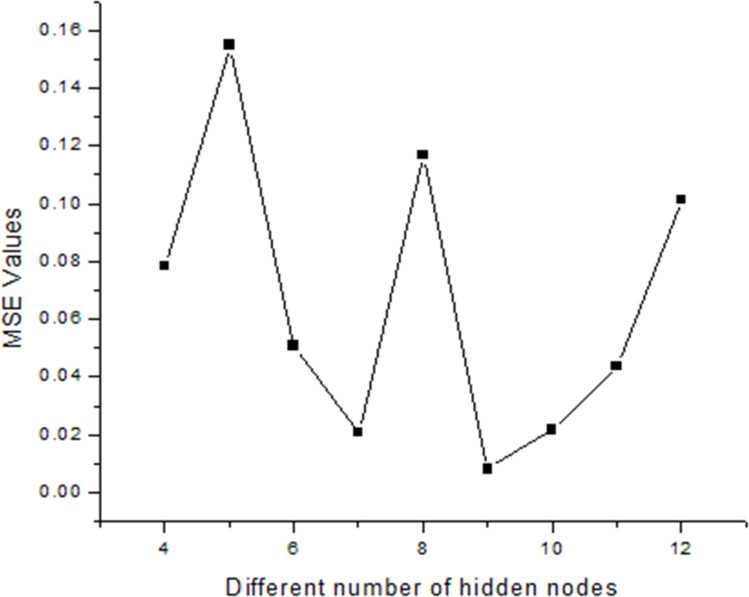
Variation of different hidden nodes with respect to their MSE values.

**Fig. 3:**
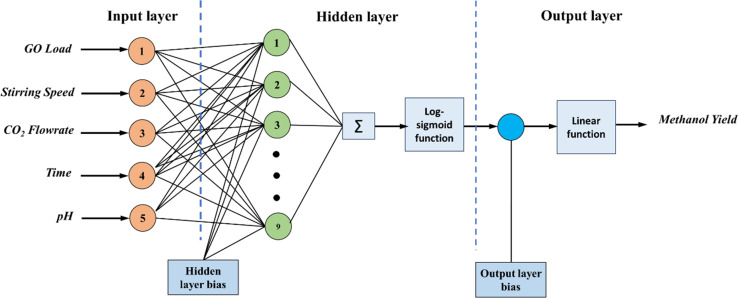
The selected ANN diagram with 5-9-1 configuration.

In the present study, a neural network architecture employing a single layer of hidden neurons was utilized, with the number of hidden neurons ranging from 4 to 12. The specific architecture employed is denoted as ANN 5-9-1, where the first digit represents the number of input nodes, ‘*N*’ signifies the variable number of hidden neurons, and the last digit indicates the number of target nodes. This configuration enables flexibility in the network's capacity to learn complex patterns and relationships within the data. During the training process, two primary metrics were utilized to gauge the network's performance and convergence: MSE values and regression (*R*) values. The MSE value quantifies the average squared difference between the predicted output and the actual target values. A lower MSE value indicates better accuracy and network performance. Conversely, the *R*-value measures the correlation between the network's output and the target values. A higher *R*-value, ranging between 0 and 1, indicates a closer relationship between the predicted and actual values, with 1 indicating a perfect correlation. The variance of maximum absolute MSE values across different numbers of hidden nodes (4–12) is depicted in Fig. 2, providing insights into the network's performance under varying complexities. This analysis offers valuable information for selecting the optimal number of hidden neurons to balance model complexity and predictive accuracy. Following thorough network evaluation, it was determined that the architecture ANN 5-9-1 exhibited the most desirable performance characteristics, boasting the lowest MSE value among the evaluated configurations. This conclusion underscores the effectiveness of the chosen neural network architecture in capturing the underlying patterns within the dataset and generating accurate predictions. The interaction of input parameters through the hidden layer of neurons has been represented in Fig. 3.

## 3. Results and discussions

### 3.1 Effects of individual parameters on methanol yield: one-factor-at-a-time

#### 3.1.1 Effects of HEG loading on methanol yield

**Fig. 4:**
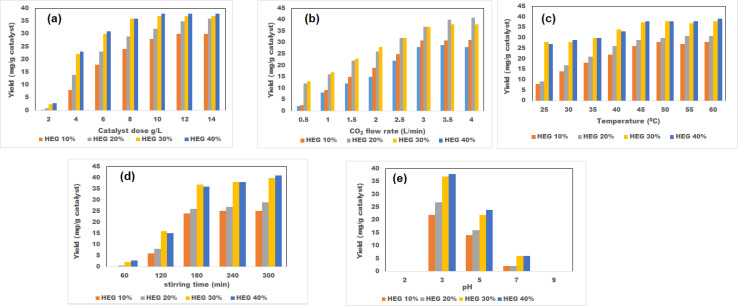
Effects of different operational conditions on methanol yield for different HEG loading of catalysts; (a) catalyst dose *vs.* methanol yield; (b) CO_2_ flow rate *vs.* methanol yield; (c) temperature *vs.* methanol yield; (d) stirring time *vs.* methanol yield; (e) pH *vs.* methanol yield.

The HEG loading in the specimens of the catalysts varied from 10% to 40% to find the optimum loading producing the best methanol yield. All the figures (Fig. 4a, c, d, and e) show that the yield of methanol is significantly higher when the HEG loading increases. This is because of the increase in HEG loading; a significant amount of CO_2_ gets absorbed by the HEG in the catalyst, which is then reduced further by the TiO_2_ in the TiO_2_-based material. Furthermore, graphene's high electron mobility on the surface makes CO_2_ reduction *via* the catalytic reaction much easier. This is why it is easily understandable that 10 wt% HEG loading produced the lowest product yields. Correlations between catalyst dose and methanol yield can be seen in Fig. 4a, where the methanol yield increases from 0.8 to 36 mg g^−1^ catalyst (HEG 20 wt%), 2.5 to 37 mg g^−1^ catalyst (HEG 30 wt%) and 2.8 to 38 mg g^−1^ catalyst (HEG 40 wt%). During the variation of the CO_2_ flow rate, the yields for different catalysts varied from 2.6 to 31.2 mg g^−1^ catalyst (HEG 20 wt%), 12 to 41 mg g^−1^ catalyst (HEG 30 wt%), and 13 to 38 mg g^−1^ catalyst (HEG 40 wt%), as shown in Fig. 4b. As per Fig. 4c. In contrast, the variation of temperature, the yields for different catalysts varied from 9.1 to 31 mg g^−1^ catalyst (HEG 20 wt%), 28 to 38 mg g^−1^ catalyst (HEG 30 wt%) and 27 to 38 mg g^−1^ catalyst (HEG 40 wt%). According to the Fig. 4d, by the increase in stirring time, methanol yields of 0.5 to 29 mg g^−1^ catalyst (HEG 20 wt%), 2.2 to 40 mg g^−1^ catalyst (HEG 30 wt%) and 2.8 to 41 mg g^−1^ catalyst (HEG 40 wt%) were obtained. A decrease in pH results in an increased methanol yield, where the 20% HEG loading resulted in a methanol yield of 27 mg g^−1^ catalyst, 30% loading produced a methanol yield of 37 mg g^−1^ catalyst and 40% loading showed a methanol yield of 27 mg g^−1^ catalyst (Fig. 4e). HEG-based catalyst containing 30 wt% HEG is observed to produce the best results in each of the cases of investigations of Fig. 4. Though a bit of difference in numerical values could be observed in 40 wt% loadings, concerning the outcome of 30 wt%, it is insignificant.

#### 3.1.2 Effects of catalyst dose on methanol yield

Methanol yield and catalyst doses of four distinct types of photocatalysts, which were differentiated by their HEG wt%, showed a positive correlation (Fig. 4a). High percentage of HEG present in the photocatalyst captures more CO_2,_ which, at the same time, leads to a higher yield of methanol. More electrons can be released at high catalyst concentrations, increasing methanol yield. At a CO_2_ flow rate of 3 L m^−1^, the pH of the solution was 3, the temperature was 50 °C, and the stirring time was 3 h, the photocatalyst dosing was varied from 2 to 14 g L^−1^. The methanol yields linearly increased for all the catalyst specimens of different HEG loadings. But, at a catalyst loading of 10 g L^−1^, the methanol yields were obtained as 32 mg g^−1^ catalyst (HEG 20 wt%), 37 mg g^−1^ catalyst (HEG 30 wt%), and 38 mg g^−1^ catalyst (HEG 40 wt%). More than this loading percentage, no significant increase in product yield could be observed for 30 wt% and 40 wt% HEG-loaded photocatalysts. Moreover, less product yield improvement (only 1 mg g^−1^ catalyst) was observed in a 10 wt% increase in HEG loading (30 wt% to 40 wt%). At very high photocatalytic doses, like 40%, it may be due to turbidity in the solution; penetration of light gets prevented inside the solution, leading to a lack of uniform light distribution on the photocatalyst active sites, which ultimately reduces the product forming reaction rates.

#### 3.1.3 Effects of CO_2_ flow rate on methanol yield

CO_2_ flow rate has a significant impact on methanol yield when using four different types of catalysts (10 wt%, 20 wt%, 30 wt%, and 40 wt% of HEG loadings) at a fixed photocatalytic dose of 10 g L^−1^, process temperature (50 °C), solution pH (3), and stirring time (3 h). As represented in Fig. 4b, methanol yields thoroughly increased when the CO_2_ flow rate was increased from 0.5 to 4 L m^−1^. Being the main raw material reactant, higher flow rates of CO_2_ can increase the conversion for methanol formation while using the HEG-loaded TiO_2_-assisted catalyst. In addition, high CO_2_ flow in the feed solution increases the methanol yield due to two factors: (i) uniform trapping of light intensity by photocatalyst and (ii) well-filled pores in the catalysts. For example, at a CO_2_ flow rate of 3 L min^−1^, yields were found as 31 mg g^−1^ catalyst (HEG 20 wt%), 37 mg g^−1^ catalyst (HEG 30 wt%), and 37 mg g^−1^ catalyst (HEG 40 wt%). Beyond this CO_2_ input rate, no significant growth in methanol yield could be observed. For methanol synthesis at high doses of HEG loading in a catalyst (*e.g.*, 40 wt%) in a solution, a lack of substrate may be to blame for low yields because of the overloading on the catalyst's active sites. Moreover, at higher flow rates of CO_2_, less residence time is needed for the effective conversion reactions that used to be provided, which causes the transformation efficiency to decline or remain constant.

#### 3.1.4 Effects of process temperature on methanol yield

Experimental investigations were run maintaining the catalyst dose of 10 g L^−1^, CO_2_ flow rate of 3 L min^−1^, pH of the solution at 3, and stirring time of 3 h. The temperature impacts methanol yield, and the results in Fig. 4c show a positive correlation with methanol yield. Linearly increased methanol yields were observed when the process temperature was increased from 40 to 60 °C, and this was due to the high frequency of collisions between the photocatalytic substrate and the feed substrate, as well as higher rates of substrate diffusion into the photocatalyst. At a temperature of 50 °C, methanol yields were found as 30 mg g^−1^ catalyst (HEG 20 wt%), 38 mg g^−1^ catalyst (HEG 30 wt%), and 38 mg g^−1^ catalyst (HEG 40wt%). No effective upsurge in product yields was observed at temperatures higher than this value. So, no change in the product yield happens even if the reaction is run with high doses of HEG loading in the catalyst (∼40 wt%) at elevated temperatures. Moreover, overheating on the catalyst's active sites further enhances the chances of the unwanted desorption effect in this process.

#### 3.1.5 Effects of stirring time on methanol yield

According to Fig. 4d, a positive correlation could be observed between stirring time and methanol yield when the stirring time was increased from 60 to 300 min, controlling the catalyst dose of 10 g L^−1^, CO_2_ flow rate of 3 L min^−1^, pH of the solution at 3 and the temperature of 50 °C. Due to the increase in stirring time, more residence time could be provided to the photocatalytic reaction system, enhancing the methanol yield. The yield of methanol per gram of catalyst was found to be very low due to incomplete hydrogenation of the feed solution up to 120 min, whereas stirring time longer than 120 min significantly increased methanol synthesis as the reaction was completed. A gradual decline in yield was observed after a relatively stable yield between 3 and 28 h of reaction time. The product (methanol) yields were found as 26 mg g^−1^ catalyst (HEG 20 wt%), 37 mg g^−1^ catalyst (HEG 30 wt%), and 36 mg g^−1^ catalyst (HEG 40 wt%) at a stirring time of 180 min. But, beyond this stirring time, an insignificant increase in the product concentration is observed; as a result, the process gets delayed with reduced productivity. As a matter of fact, due to the gradual loss of catalytic activity, regeneration of the catalyst became a necessity.

#### 3.1.6 Effects of solution pH on methanol yield

As shown in Fig. 4e, the effects of solution pH are inversely proportional to the methanol yield. The pH was varied from 2 to 9, keeping the constant values of other parameters like 10 g L^−1^ catalyst dose, 50 °C process temperature, 3 L m^−1^ CO_2_ flow rate, and 3 hours of stirring time. The investigation was unsuccessful at a low pH value of 2, where the yield decreases above pH 3. The product yields were found as 27 mg g^−1^ catalyst (HEG 20 wt%), 37 mg g^−1^ catalyst (HEG 30 wt%), and 38 mg g^−1^ catalyst (HEG 40 wt%) by adjusting pH 3 of the solution. This increase in methanol yield is attributed to increased carbonate ions (CO_3_^2−^) generation as the experimental investigations were run at higher pH levels than pH 3.0. Beyond this pH range, the adaptation of photo-generated electrons by the CO_3_^2−^ ions significantly decreases, preventing the formation of methanol through hydrogenation.^4^

### 3.2 Statistical analysis through ANOVA

During the development of the regression model, all five parameters (GO load, stirring speed, CO_2_ flow, time, and pH) were considered input variables, and methanol yield (%) as the output or response variable. The statistical coefficients were generated using Design Expert Software 11.0, as shown in eqn (6).

6 Observed methanol yield (%) = 38.315 + 4.075*A* + 0.275*B* + 1.025*C* − 0.925*D* + 2.025*E* − 1.59375*AB* + 0.59375*AC* + 1.40625*AD* + 1.15625*AE* − 0.78125*BC* − 0.71875*BD* + 2.03125*BE* − 0.15625*CD* − 1.03125*CE* + 0.53125*DE* − 4.39375*A*^2^ − 6.14375*B*^2^ − 1.89375*C*^2^ − 1.39375*D*^2^ − 6.76875*E*^2^

The analysis of variance (ANOVA) approach was used to determine the significant parameters in the regression model equation, which was then utilized to validate the equation. A study of numerous outcomes was conducted using Fisher's statistical test (*F*-test), a statistical approach applied. When the value of *F* is greater than the corresponding coefficient value, the value of *P* is notable. When the value of *P* is smaller than the corresponding coefficient value, the value of *F* is exceptional. When the sum of squares value is significantly greater than the mean, it is noticed that the importance of any process variable is also much larger. To get methanol yield, the value of 61.98 must be used in conjunction with the equivalent value of less than 0.0001, the sum of squares value of 4786.74, the value of 20, and the mean square value of 239.34. When the *F*-value of a model exceeds zero, it is considered significant. An *F*-value this large is around a 0.01% probability of being caused by random noise. A model term's *P*-value is less than 0.05 and considered significant. Greater or equal to 0.1000 values suggest model terms have little significance. The number of meaningless terms in the model can be reduced by eliminating those that are not necessary (such as those needed to maintain hierarchy). A statistically significant lack of fit is indicated by an *F*-value of 40.40. There is a 0.01% chance that the high lack of fit *F*-value is caused by noise.

The model was tested with a wide range of process parameters for the best methanol yield using ANOVA. Using the model's standard deviation of 1, mean of 21, and CV percent value of 9, the model has a correlation coefficient of 97.72%, indicating good model accuracy. Furthermore, 96.14 and 91.45 are the respective models that predicted and observed *R*^2^ found in ANOVA. The desirability index from the design experiment was used to evaluate the model's applicability under different process conditions. Maximum methanol yield of up to 36.3 mg g^−1^ catalyst was achieved under the optimized governing conditions: HEG loading of 30% and stirring speed of 200 rpm, the CO_2_ flow rate of 3 L min^−1^, stirring period of 90 min, and pH at 3. According to the attractiveness index, the model is well-suited for analyzing the design experiment under discussion.

**Fig. 5:**
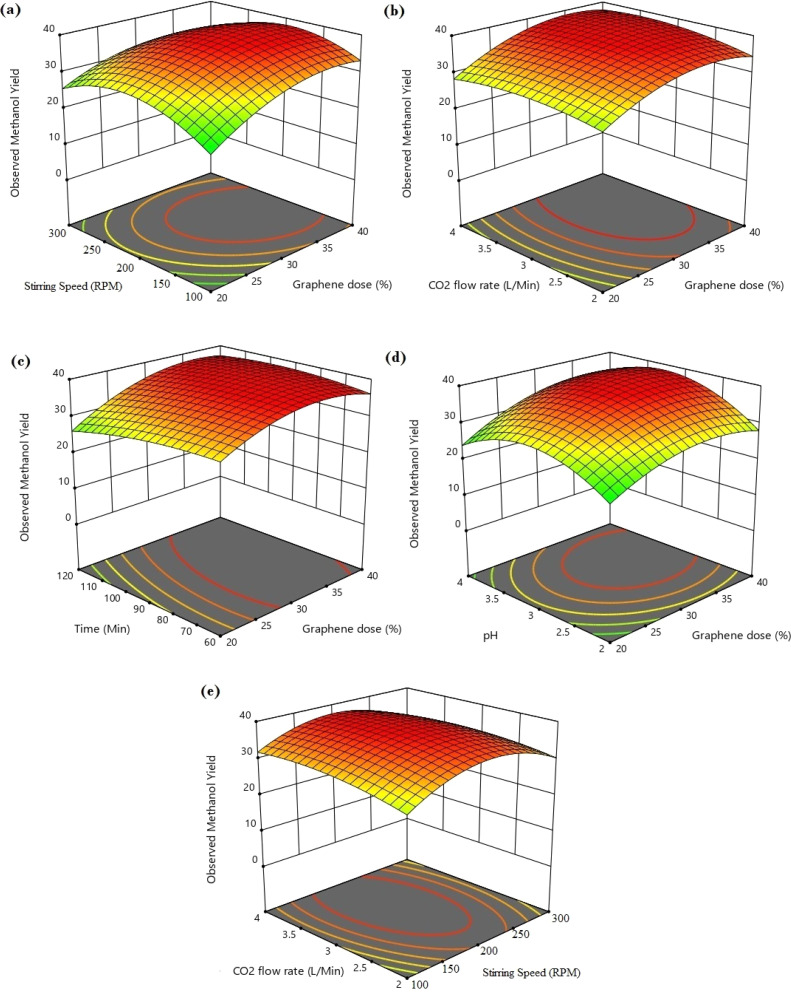
3D type conjugate diagram of methanol yield at different operating conditions; (a) stirring time and graphene dose *versus* observed methanol yield; (b) CO_2_ flow rate and graphene dose *versus* observed methanol yield; (c) time and graphene dose *vs.* observed methanol yield; (d) pH and graphene dose *versus* observed methanol yield; (e) CO_2_ flow rate and stirring speed *versus* observed methanol yield.

**Fig. 6:**
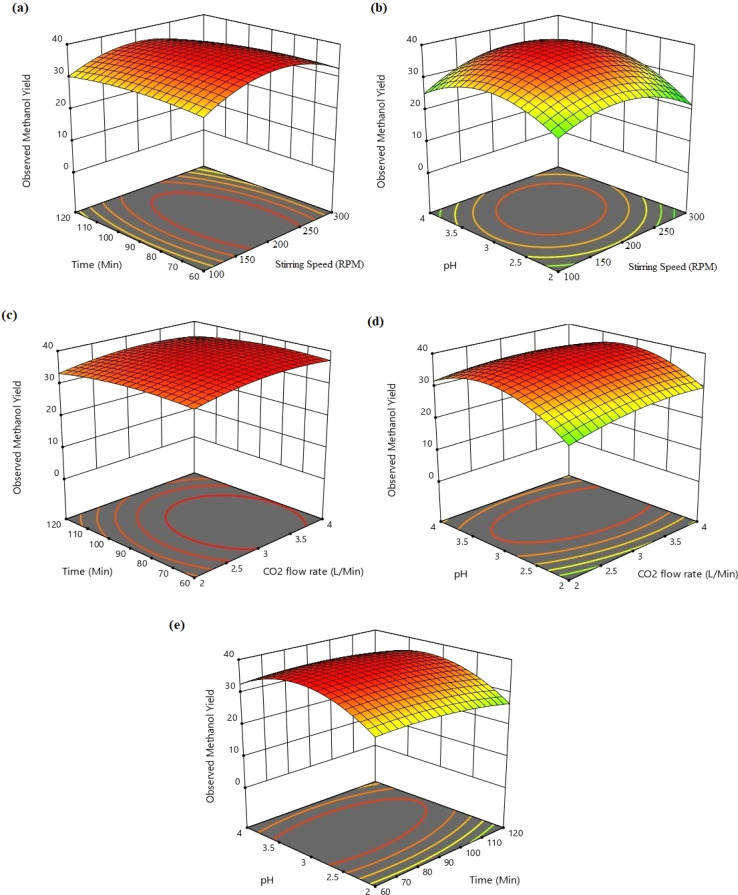
3D type conjugate diagram of methanol yield at different operating conditions; (a) time and stirring speed *versus* observed methanol yield; (b) pH and stirring speed *vs.* observed methanol yield; (c) time and CO_2_ flow rate *versus* observed methanol yield; (d) pH and CO_2_ flow rate *vs.* observed methanol yield; (e) pH and time for observed methanol yield.

The different conjugate diagrams of observed methanol yield using various input parameters have been shown in [Fig. 5a–e and 6a–e]. The actual data originated from the experimental runs involving all process input parameters (GO load, stirring speed, CO_2_ flow, time, and pH), as shown in the CCD (Table 2). The software calculates the predicted values with the help of mathematical model equations. The correlation coefficient (*R*^2^) value is measured using actual and predicted values. The conjugate plot of graphene oxide load and stirring speed on product yield has been depicted in Fig. 5a. Observing methanol yield increased from 25 to 32 mg g^−1^ of catalyst when GO load was increased from 20% to 40%, and stirring speed was increased from 100 to 300 rpm. Simultaneous effects of CO_2_ flow rate and graphene oxide load have been shown in Fig. 5b, where the observed methanol yield got enhanced from 28 mg g^−1^ catalyst to 36 mg g^−1^ catalyst when graphene oxide load and CO_2_ flow rate increased from 20% to 40% and from 2 to 4 L min^−1^, respectively. The coupled effects of time and GO load have been represented in Fig. 5c. This shows that the observed methanol yield (%) increased from 25 to 36 mg g^−1^ of catalyst when the time was increased from 60 to 120 min, and the graphene oxide load increased from 20% to 40%. The conjugate diagram of pH and GO load has been revealed in Fig. 5d. When pH and GO load were increased from 2 to 4 and 20% to 40%, respectively, methanol yield was enhanced from 22 to 30 mg g^−1^ of catalyst. Simultaneous effects of CO_2_ flow rate and stirring speed have been shown in Fig. 5e, where the observed methanol yield increased from 8 to 30 mg g^−1^ of catalyst when the CO_2_ flow rate was increased from 2 to 4 L min^−1^ and stirring speed was increased from 100 to 300 rpm. The coupled effects of time and stirring speed have been depicted in Fig. 6a. It could be seen that the methanol yield upsurged from 8 to 33 mg g^−1^ of catalyst when time increased from 60 to 120 min, and stirring speed increased from 100 to 300 rpm. The conjugate effects of pH and stirring speed have been represented in Fig. 6b. Observed methanol yield grew from 0 to 25 mg g^−1^ catalyst when pH increased from 2 to 4, and catalyst dose increased from 100 to 300 rpm. Simultaneous effects of time and CO_2_ flow rate have been shown in Fig. 6c. Observed Methanol yield enhanced from 12 to 32 mg g^−1^ of catalyst when flow rate increases from 2 to 4 L min^−1^ and time increases from 60 to 120 min. The conjugate effects of pH and CO_2_ flow rate have been revealed in Fig. 6d. Methanol yield has been observed to rise from 0 to 32 mg g^−1^ catalyst when pH rises from 2 to 4 and CO_2_ flow rate increases from 2 to 4 L min^−1^. Simultaneous effects of pH and time have been shown in Fig. 6e. Methanol yield has been observed to increase from 8 mg g^−1^ catalyst to 35 mg g^−1^ catalyst when pH increases from 2 to 4 and time increases from 60 to 120 min. Leonzio, investigated the design of experimental analysis using RSM to analyze the synthesis of methanol for better process efficiency.^25^ They have found that carbon conversation, methanol yield, methanol selectivity, and methanol production are higher than 60%, higher than 60%, between 90% and 95%, and higher than 0.15 mol h^−1^, respectively, with a feed flow rate of 1 mol h^−1^. Rafiee also performed modeling and optimization of methanol synthesis from hydrogen and CO_2_ through RSM and ANOVA.^24^ They noticed a 95% correlation coefficient obtained at a flow rate of 5 L min^−1^, pH of 6, and time of 30 min. ANOVA analysis validated the model with different process parameters for maximum methanol yield.

### 3.3 Statistical analysis through ANN

**Fig. 7:**
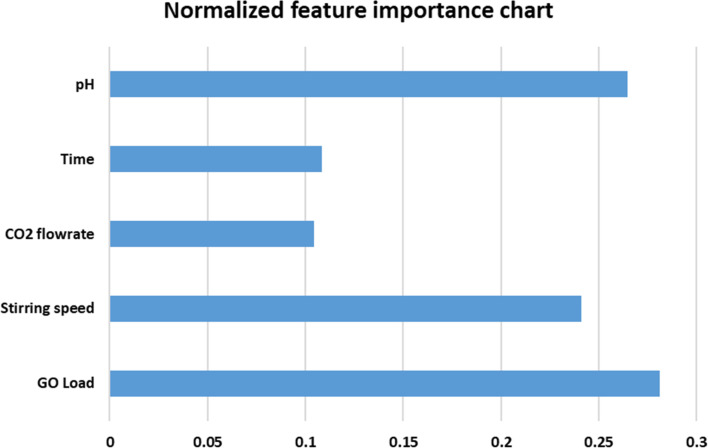
Normalized feature importance chart.

Fig. 7a and b depict the model validation profiles of the ANN model when applied to the experimental database. In contrast, Fig. 6 depicts the regression curve for each training, testing, and validation data set. In the depicted ANN architecture, as shown in Fig. 3, extensive experimentation has revealed that achieving optimal performance is contingent upon several key architectural decisions. Specifically, after rigorous testing and analysis, it was determined that the ideal configuration involves employing a total of 9 neurons. Furthermore, the activation functions utilized within the hidden and output layers play a pivotal role in shaping the network's efficacy. Through meticulous evaluation, it was established that the combination of the log-sigmoid activation function within the hidden layers, complemented by the linear activation function in the output layer, consistently yields superior performance across various metrics and tasks. This strategic choice in activation functions enables the network to effectively capture and propagate complex nonlinear relationships within the data, resulting in enhanced predictive accuracy and generalization capabilities. The overall regression score of 0.988 showcases the exceptional performance of the ANN model in accurately capturing the underlying patterns within the dataset. This high score signifies that the model has proficiently completed the fitting process, effectively encapsulating the complexities of the design experiment. To elaborate on this technical aspect, the regression score, also known as the coefficient of determination (*R*^2^), quantifies the proportion of the variance in the dependent variable that is predictable from the independent variables. A score of 0.988 implies that approximately 98.8% of the variability in the target variable is explained by the input features utilized in the ANN model. Such a remarkable score underscores the robustness and efficacy of the model in capturing the intricate relationships present in the data.

To compare the ANN prediction and existing equations based on the experimental database, the ratio *M*_test_/*M*_calc_ was used.

*M*_calc_ = predicted value of methanol yield.

*M*_test_ = experimental value.

A statistical analysis was performed to evaluate the ANN model with experimental values, as shown in Table 2. It was necessary to analyze the standard deviation (SD), mean, and coefficient of variation (COV) to determine how well the ANN results tracked the experimental data in the database. The presence of a mean value close to one with a slight standard deviation indicates that the ANN network can generalize the information. The coefficient of variation was used to determine the precision of the findings obtained by employing the ANN model, and it was calculated as follows: it depicts the degree to which variability varies in proportion to the mean value. A lower coefficient of variation indicates that the amount of scattering in the data has been reduced to a minimum. The mean, standard deviation, and coefficient of variance are determined using the experimental/predicted ratio, and they are as follows: 1.010151, 0.115257, and 11.409%. In Fig. 7, a normalized feature importance chart is presented, providing a comprehensive visual representation of the relative significance of each feature. This visualization facilitates a deeper understanding of the impact that individual features exert on the overall outcome or phenomenon under investigation. This insight aids in prioritizing resources and efforts towards the most impactful features, thereby optimizing decision-making processes and enhancing the efficacy of subsequent actions. The normalization of feature importance ensures a fair comparison across different features, mitigating biases stemming from variations in scale or measurement units. This enables researchers, analysts, and practitioners to make informed judgments regarding feature selection, model refinement, and strategic planning, fostering more robust and insightful analyses.

**Fig. 8:**
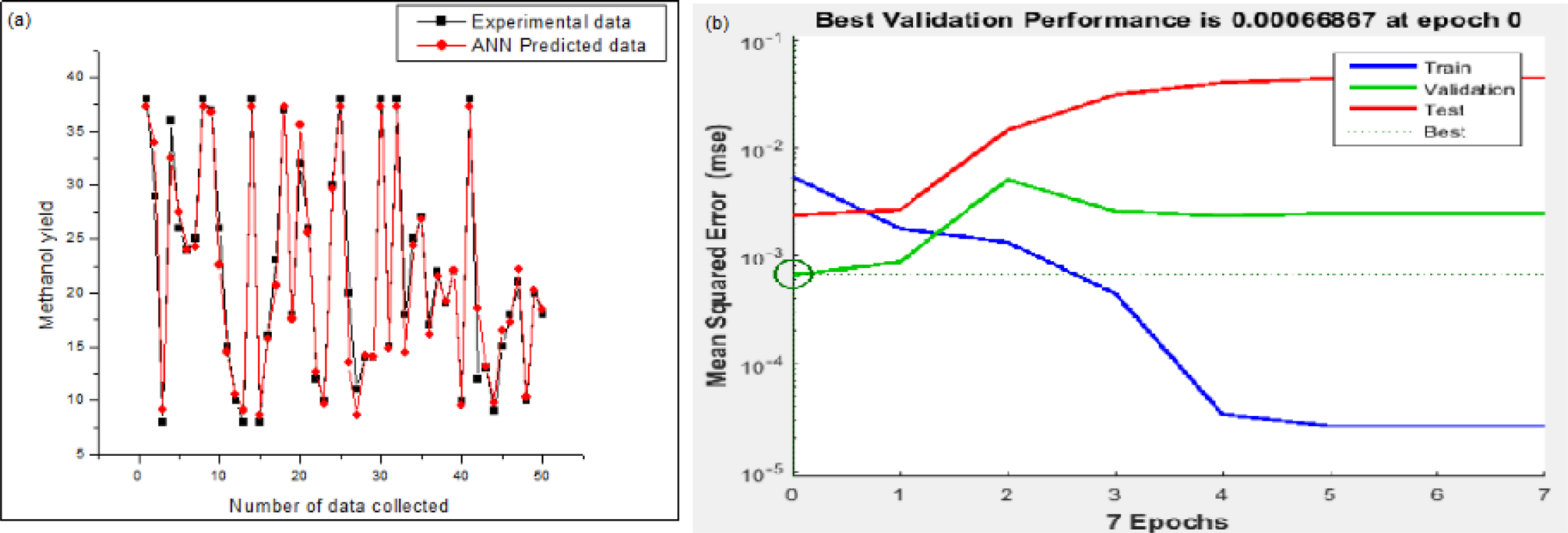
Model validation results of ANN: (a) experimental *versus* predicted output of ANN model; (b) performance graph of ANN.

**Fig. 9:**
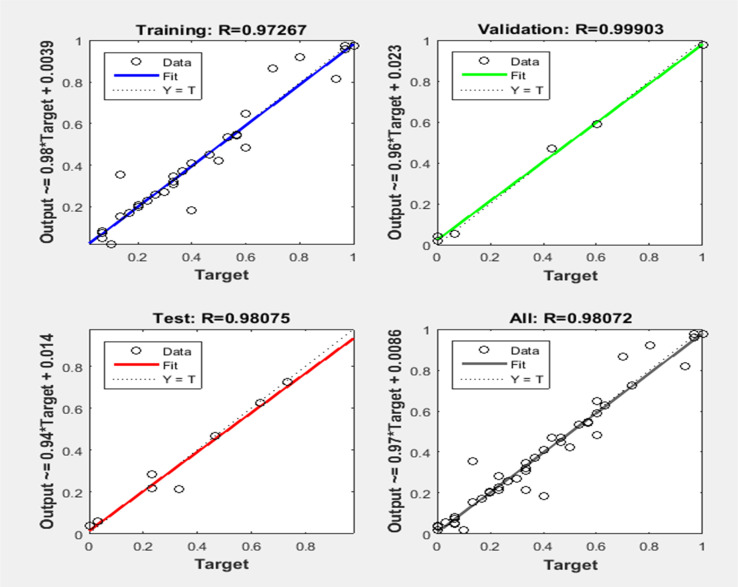
The regression curves of the ANN model 5-9-1.

As per Fig. 8a, a reasonably good corroboration could be observed between the experimentally obtained methanol yield data concerning the predicted values of ANN. The performance graph of the epochs value and the MSE for the ANN model utilizing these input parameters is depicted in Fig. 8b. It can be observed from the graph that the best validation performance is 0.00066867 at epochs 0 and 1. A representation of the training, testing, and validation of these distinct epochs in terms of MSE values may be found in Fig. 8b. A similar outcome has been seen by several researchers who have carried out methanol synthesis from CO_2_ and hydrogen gas utilizing ANN modeling throughout the last few years. Also, in the case of Fig. 9, well corroborations in data fitting for this 5-9-1 ANN model could be observed with high values of regression coefficients. The ANN modeling of syngas to methanol production and other topics were investigated in the literature.^37^ According to the findings of this research study, an ANN model has been constructed to simulate the generation of methanol from fuel gas. Overall, the results showed that raising pressure from 10 to 30 bar increased methanol concentration and CO conversion, which climbed from 21.1 percent to 22.3 percent and from 34.0 percent to 36.2 percent, respectively, with the increase in pressure. A kinetic investigation of carbon dioxide conversion to methanol over innovative carbon nanofiber-based Cu/ZrO_2_ catalysts was carried out by Uddin *et al.*, who then verified their findings with the use of an ANN.^10^ The results of an ANN were found to be validated in terms of an experimental kinetic analysis. Chuquin-Vasco *et al.* used neural networks to estimate methanol output in a carbon dioxide hydrogenation plant, using 133 data pairs and 12 hidden neurons in their analysis.^38^ The reported values for the global regression coefficient *R*^2^ and the global mean square error (RMSE) were 0.9442 and 0.0085, respectively.

### 3.4 Optimization of ANN model using genetic algorithm (GA)

In our study, we leveraged the capabilities of ANN models, which, owing to their inherent nature as black boxes, were utilized for optimization purposes. Specifically, we employed a Genetic Algorithm (GA) as the optimization technique. The parameters under consideration—HEG loading, stirring speed, CO_2_ flow rate, reaction time, and pH—were bounded within ranges to ensure practical feasibility. These bounds were set as follows: HEG loading ranged from 10% to 50%, stirring speed from 100 to 300 rpm, CO_2_ flow rate from 1 to 5 L min^−1^, reaction time from 30 to 150 min, and pH from 1 to 10. Through this comprehensive approach, we successfully achieved an optimal methanol yield of 37.81 mg g^−1^ catalyst. This significant outcome was obtained under specific conditions: HEG loading at 30.16%, stirring speed of 201.25 rpm, CO_2_ flow rate of 2.96 L min^−1^, reaction time of 90.21 min, and pH of 2.86. Remarkably, upon closer examination, these optimal conditions closely resemble those initially identified in the first run of our experimental design matrix, as illustrated in Table 2. This convergence not only validates the effectiveness of our optimization methodology but also underscores the robustness of our experimental setup.

## 4. Error analysis

Errors calculated during the validation of model-predicted data with experimental data are a good indicator of model development. However, in the past, only a few standard statistical methods were used to evaluate errors in the model data. Therefore, the accuracy of the model's predictions compared to the experimental data was examined using the statistical tools discussed below.

### 4.1 Relative error through RMSE analysis

For the calculation, RMSE (Root Mean Square Error), the following expression has been used:
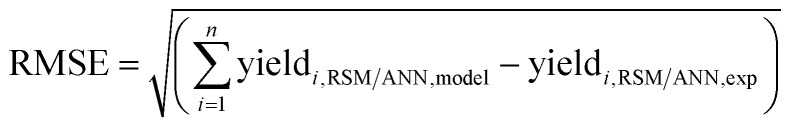
where yield_*i*,RSM/ANN,model_ and yield_*i*,RSM/ANN,exp_ are the respective outcomes of model and experimental investigations extracted from RSM or ANN optimization study, *i* is the number of runs.

The following expression can be used to calculate the relative error (RE) from the average of the experimental data (yield_*i*,(RSM/ANN,exp)avg_):RE = (RMSE/yield_(*i*,RMS/ANN,exp)avg_)

### 4.2 *d*-Willmott index (*d*_Willmott_)

The *d*-Willmott index (*d*_Willmott_) parameters were calculated using the equation below:
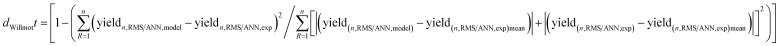


### 4.3 Regression coefficient (*R*^2^)

*R* ^2^ was determined with the help of the following equation:
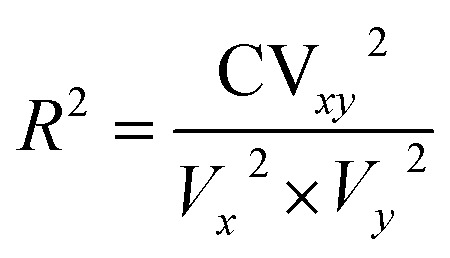
where, CV_*xy*_^2^ = the covariance of the dependent and independent variable, *V*_*x*_^2^ = variance of the dependent variable, *V*_*y*_^2^ = variance of the independent variable.

Suppose the regression coefficient is >98%. In that case, the relative error is less than 0.09, and the *d*_Willmott_ is greater than 95% for the experimental outcome, a significant model prediction is nominated. Table 3 shows the results of calculating the yield data's error values to assess the newly developed mathematical approach.

**Table 3:** Comparative results of statistical analysis of RSM and ANN model data

Optimization techniques	Statistical analysis			
	Responses	*R* ^2^	Relative error	*d*_Willmott index_
RSM	Yield_RMS,exp_*vs.* yield_RMS,model_	0.972	0.11	0.950
ANN	Yield_ANN,exp_*vs.* yield_ANN,model_	0.988	0.054	0.977

## 5. Conclusions

It appears that, against the backdrop of several established and emerging CO_2_ capture and conversion technologies, graphene-coated catalysts produce a high yield of methanol from CO_2_. However, most procedures fail during critical decision-making based on all of the elements where ANN and RSM methods can perform effective model prediction. Therefore, the two methods were compared to determine which produced the highest yield. According to the ANOVA table, HEG weight percentage, CO_2_ flow rate, stirring speed, temperature, stirring time, and solution pH had major effects on the yield quantity. The most significant parameters identified to control the reaction mechanism were HEG dosing and volumetric input rate of CO_2_. 30 wt% HEG loading in developed photocatalyst provided the highest methanol yield of 36.3 mg g^−1^ catalyst, was achieved under the other optimized governing conditions, such as stirring speed of 200 rpm, the CO_2_ flow rate of 3 L min^−1^, stirring period of 90 min, and pH at 3. Correlation coefficients for both methods (ANN and RSM, *R*^2^ = 0.985 and *R*^2^ = 0.971, respectively) indicate that the ANN model is better predictive and more accurate than the RSM model. However, the RSM-proposed model is equally capable of predicting the real findings. According to the results, the optimal point of methanol yield for the RSM and ANN models was 36.3 and 37.3 mg g^−1^ of catalyst, respectively.

## Conflicts of interest

There are no conflicts to declare.

## Supplementary Material
